# Development of personal air pollution exposure report-back materials to Household Air Pollution Intervention Network (HAPIN) trial participants in Guatemala and Rwanda: a qualitative study

**DOI:** 10.1136/bmjgh-2024-017672

**Published:** 2025-05-15

**Authors:** Ashlinn Quinn, Mayari Hengstermann, Anaite Diaz-Artiga, Ajay Pillarisetti, Maggie Clark, Laura Ruiz-Aguilar, Florien Ndagijimana, John P McCracken, Ghislaine Rosa, William Checkley, Jennifer Peel, Thomas F Clasen, Lisa Thompson, HAPIN Investigators, Vigneswari Aravindalochanan

**Affiliations:** 1 Berkeley Air Monitoring Group, Fort Collins, California, USA; 2 Universidad del Valle de Guatemala, Guatemala, Guatemala; 3 Environmental Health Sciences, University of California Berkeley, Berkeley, California, USA; 4 Colorado State University, Fort Collins, Colorado, USA; 5 Graphic Designer, Guatemala, Guatemala; 6 Eagle Research Center, Kigali, Rwanda; 7 University of Georgia, Athens, Georgia, USA; 8 University of Liverpool, Liverpool, UK; 9 Division of Pulmonary and Critical Care, Johns Hopkins University, Baltimore, Maryland, USA; 10 Environmental & Radiological Health Sciences, Colorado School of Public Health, Aurora, Colorado, USA; 11 Gangarosa Department of Environmental Health, Emory University, Atlanta, Georgia, USA; 12 Nell Hodgson Woodruff School of Nursing, Emory University, Atlanta, Georgia, USA

**Keywords:** Global Health, Pneumonia, Qualitative study

## Abstract

**Background:**

Report-back of individual exposure information to research participants is recognised in high-income countries as an important, yet often overlooked, component of environmental research, with many potential benefits to study communities. Nonetheless, the optimal means of communicating findings to participants in low-income countries with limited health and scientific literacy is unknown.

**Methods:**

Between March 2021 and May 2022, we conducted a qualitative study with 61 women and 20 of their household members (n=81) participating in the Household Air Pollution Intervention Network trial in Guatemala and Rwanda. Using participant observations and individual interviews (n=61), group interviews (n=21), dynamic working groups (n=78) and focus groups (n=45), we collaborated with study participants to iteratively develop contextually appropriate and comprehensible materials that conveyed individual air pollution exposures.

**Results:**

Posters were generated to display graphical representations of participants’ personal air pollution exposures, along with the known health effects of air pollution exposure and actions that could be taken to reduce their exposures to household air pollution.

**Discussion:**

This is the first study to report back personal household air pollution exposure results to study participants in two low-income countries where people rely on biomass fuel (eg, wood, crop waste, dung) for cooking. We used community-engaged methods to co-produce locally and contextually specific materials.

**Trial registration number:**

NCT02944682.

**WHAT IS ALREADY KNOWN ON THIS TOPIC**

- While considerable progress has been made documenting methods and tools that can be used to present personal study results to participants of scientific research conducted in high-income countries, to our knowledge, there has been no documented research on this issue in low-income research settings.

**WHAT THIS STUDY ADDS**

- We used community-engaged methods to develop and test graphical displays of personal environmental data and educational materials that were comprehensible for participants with limited literacy in rural Guatemala and Rwanda.

**HOW THIS STUDY MIGHT AFFECT RESEARCH, PRACTICE OR POLICY**

- Informing research participants about their exposures is an ethical obligation; doing so enhances study participants’ capabilities to modify behaviours that may protect health.

## Introduction

 In the context of studies that measure exposures to environmental pollutants, reporting back individual data to research participants is an important aspect of the science. Report-back of personal exposure data to participants aligns with the justice pillar of the Belmont Report, where those who bear the risks of scientific research should, whenever possible, be among the first to benefit from its insights.^1^ Report-back comes from a desire to respect research participants’ contributions to science, viewing study participants as active partners in the research who also want to know their individual data. Informing research participants about their exposures to pollutants can lead to enhanced participant engagement and allow participants to make informed decisions about reducing their exposures.^1 2^

Routine report-back of findings is gaining traction, as evidenced by guidance from a number of American, European and Canadian health research agencies (see table 1 in Brody *et al*^2^ for a list of examples). As a 2018 National Academies of Sciences, Engineering and Medicine report^3^ suggests, this trend aligns with “the larger cultural transition toward more engagement, collaboration, and transparency between investigators and research participants” (pageix, preface). While the return of certain research results, such as genetic information, has become much more widespread in recent years,^4^ fewer studies have addressed the return of non-genetic results to participants.^5^ Studies pertaining to environmental pollutants, notably, fall into a category of research where “implementation of environmental health report-back practices is not yet routine”.^6^ Moreover, most publications regarding report-back focus on research conducted in high-income countries.^5^,^8^ The scientific rationale for the present study is that, although some environmental health research has focused on marginalised and minority populations in the USA, such as migrant farmworkers,^9^ the Dine (Navajo people)^10^ and low-income parents of children with asthma,^11^ evidence documenting individual report-back of exposure results in low- and middle-income countries is non-existent to our knowledge.

The objectives of this exploratory, qualitative study are to develop contextually appropriate and comprehensible report-back materials describing personal air pollution exposure results to participants of the multi-country Household Air Pollution Intervention Network (HAPIN) trial, and to assess study participants’ environmental health literacy, defined as “an understanding of the connection between environmental exposures and human health”.^12^ The HAPIN trial (www.hapintrial.org, ClinicalTrials.gov identifier NCT02944682), conducted between May 2018 and September 2021, was a randomised controlled trial of a liquefied petroleum gas (LPG) stove-and-fuel intervention provided to 3195 pregnant women in four countries: Guatemala, India, Peru and Rwanda. Pregnant women were enrolled if they were 18 to <35 years old, had a singleton pregnancy between 9 and <20 weeks of gestation confirmed by ultrasound, and predominantly cooked with a biomass (eg, wood, crop waste, dung) stove. Women who reported smoking were excluded. Women assigned to the intervention group received a free LPG stove and unlimited fuel and were followed for 18 months.^13^ The study settings were selected based on a combination of factors, including burden of disease attributable to air pollution, history of prior household air pollution research, rural environment and providing a diversity of participants from different global south locations; further details of the study design can be found in the protocol paper.^13^ The four primary outcomes of the trial were: infant birth weight,^14^ stunting^15^ and severe pneumonia,^16^ and blood pressure in adult women residing in the pregnant women’s home. We conducted personal exposure to air pollution at three time points during pregnancy, at baseline (9–20 weeks of gestation) and two times after randomisation (between 24–32 and 32–36 weeks of gestation, respectively). We measured 24-hour fine particulate matter (PM_2.5_), using the RTI Enhanced Children’s MicroPEM (ECM, RTI International).^17^ These lightweight devices were worn by women in customised vests or aprons. The HAPIN trial continued to follow the cohort of children through 5 years of age in Guatemala, Rwanda and India.

The main HAPIN trial protocol was designed to provide aggregate-level trial results to communities at the conclusion of the study, in the form of group presentations on the study’s primary scientific findings. Because individual report-back of air pollution exposure results was not an activity originally planned for the trial, we obtained a supplement (NIH NOT-OD-20-038: Administrative Supplement for Research on Bioethical Issues) to develop materials to meet this goal. Of the four HAPIN sites, Guatemala and Rwanda lacked infrastructural and policy support for access to LPG.^18 19^ Thus, we chose to work with subsets of HAPIN participants in these two countries to optimise strategies that participants, HAPIN communities and local policy makers could take to promote the use of cleaner cooking fuels in the post-trial period.

## Methods

### Study setting and overview of study design

Between March 2021 and May 2022, we recruited a subsample of HAPIN study participants in 17 communities in Santa Maria Xalapán, Jalapa, Guatemala and four districts in Kayonza, Rwanda. In Guatemala, a list of 50 women (25 from the intervention arm; 25 from the control arm) was generated by the local principal investigator, of which 41 consented to participate (82% response rate). Participants who had completed the HAPIN trial and were currently enrolled in the follow-up cohort, as well as those who were still active in the trial, were invited to attend. In Rwanda, the field project manager invited a convenience sample of 20 participants, 10 in each study arm. In Jalapa, Guatemala, Spanish is spoken by 99% of inhabitants.^20^ The national language, spoken by all Rwandans, is Kinyarwanda.^21^ Thus, there were no exclusions to participation based on a second language spoken.

All field activities were led by a Guatemalan anthropologist (MH). Participants were recruited and consented by local, trained fieldworkers in their native language during a routine visit to the household. In Guatemala, the anthropologist, together with a team of local project staff, collected the data. In Rwanda, the anthropologist trained and supervised the local Rwandan team in qualitative research techniques. They then led the data collection in Kinyarwanda.

### Environmental literacy survey

Environmental health literacy is increasingly understood as important to research translation in environmental health research, with tangible benefits to communities and for environmental justice.^12^ Although a complex field, environmental health literacy starts “when an individual understands the link between environmental exposures and health outcomes”.^22^ In March 2021, a brief, 16-question quantitative survey in Spanish was conducted among 37 of the 41 HAPIN participants in Guatemala to assess their environmental health literacy (see online supplemental S1). The survey assessed three aspects of environmental health literacy: *Awareness and knowledge* (of environmental exposures and their effects on health), *Skills and self-efficacy* (ability to enable health-protective decisions) and *Community change* (collective action to reduce exposures and protect health).^23^ The survey was not deployed in Rwanda due to time constraints.

### Design of report-back materials

**Figure 1:**
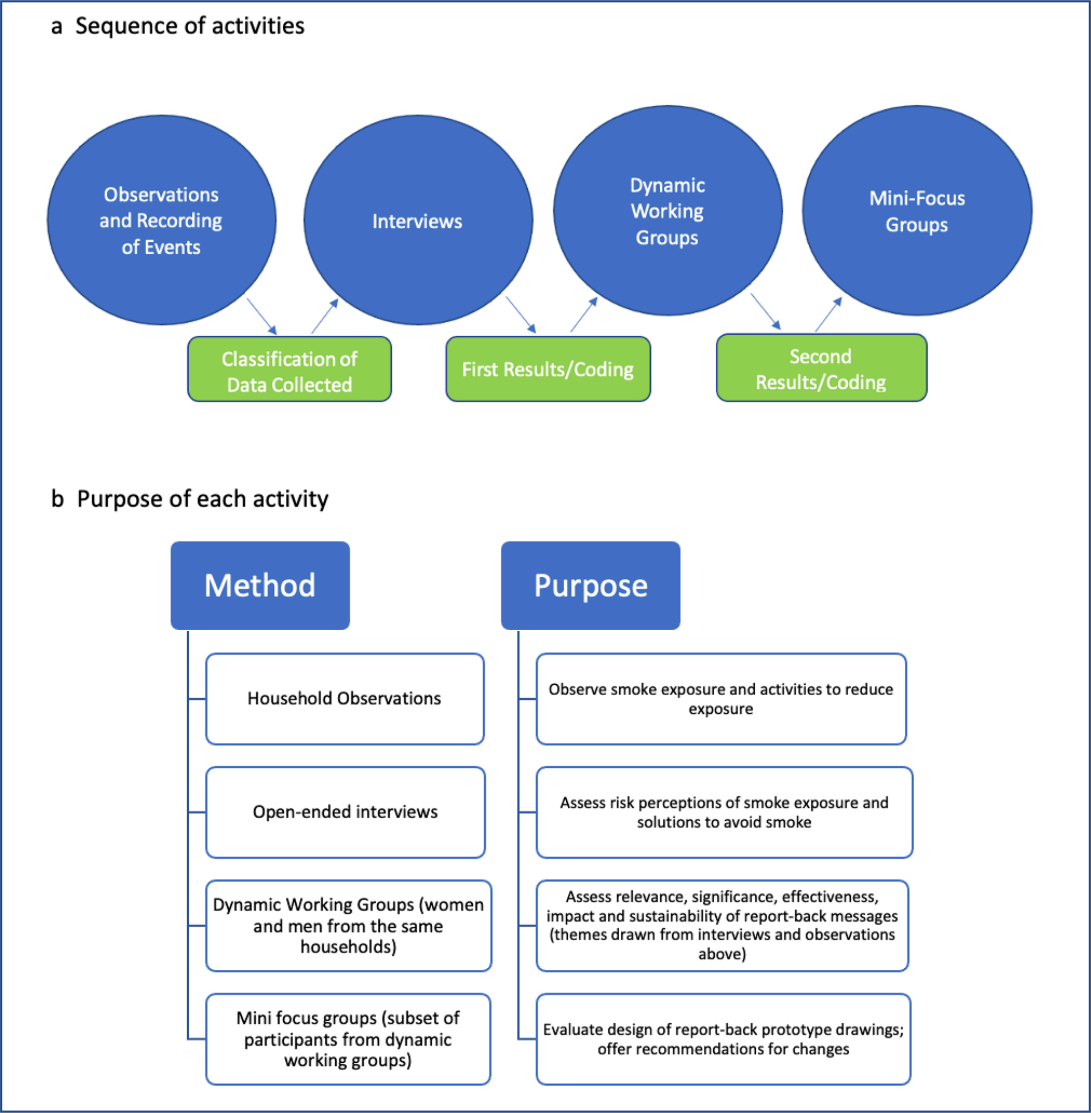
Overview of qualitative methods.

We worked with HAPIN participants in Guatemala and Rwanda to develop contextually appropriate and accessible materials that presented information to study participants about their personal air pollution exposures. In accordance with existing recommendations for reporting personal exposure results,^9^ we developed materials that combined text and graphs, used comparisons with other participants and with global guideline values to contextualise exposure findings, and included information about how participants might realistically reduce exposures. Using qualitative methods outlined in steps 1–4 and figure 1, report-back materials were iteratively prototyped, field-tested, co-designed through participant engagement and refined to ensure resultant materials were easily understandable, actionable and provided setting-specific information.

*Step 1: Participatory observations*. We conducted household participatory observations to gather data on the context and performance of cooking activities in 61 households (41 in Guatemala; 20 in Rwanda). In both Guatemala and Rwanda, observations were limited to one main meal. Our observations focused on food preparation, cooking time, stove use, appearance of cooking area (eg, smoke, ventilation) and other activities that participants engaged in while preparing food, such as caring for their children and other household chores. These observations allowed us to evaluate stove performance, elucidate the advantages and disadvantages of cooking with LPG and/or biomass, and understand how different stoves are used depending on the type of food that is being prepared. In Guatemala, an anthropologist and a fieldworker conducted the observations. In Rwanda, fieldworkers were trained by the anthropologist to conduct these observations. They attended a workshop via Zoom and received didactic materials before conducting the participant observations. Weekly Zoom meetings were held with the anthropologist to clarify questions and discuss findings.

*Step 2: Open-ended individual interviews and group interviews*. After the participatory observations, we conducted open-ended individual interviews with 41 participants in Guatemala and group interviews with 20 women in Rwanda. We explored participants’ perceptions of the health impacts of smoke exposure, their practices related to household air pollution exposures, and their opinions regarding the advantages and disadvantages of their cookstoves (LPG and/or biomass). To reduce bias, interviews were conducted until we reached data saturation.

*Step 3: Dynamic working groups (DWGs)*. We conducted DWGs with these same participants, some of whom brought their partners. In Guatemala, we conducted six DWGs, three intervention groups and three control groups. In total, 54 people participated in the activities, 39 women (21 intervention; 18 control) and 15 men (six intervention; nine control). In Rwanda, we conducted eight DWGs, in which 24 female participants, representing both control and intervention groups, worked together.

**Figure 2:**
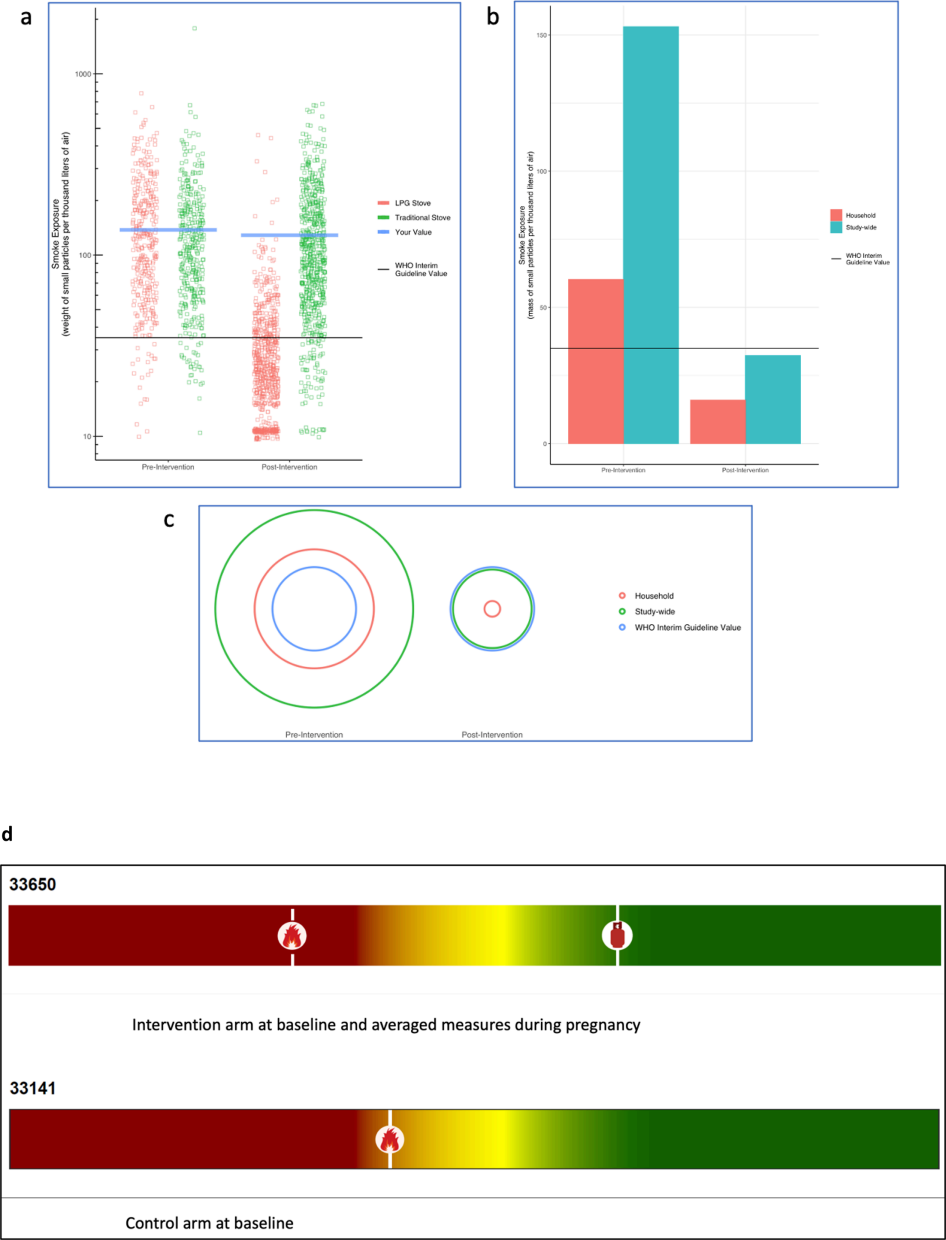
Examples of graphical displays of PM_2.5_ exposure data presented to dynamic working groups. Script read to participants, “These are displays of results from the Household Air Pollution Intervention Network study. The displays show the amount, or level, of particulate matter you were exposed to during the study. Particulate matter is a component of smoke and it is measured in terms of its mass, or weight, per certain amount of air. In this case, we measure it in terms of micrograms of PM per thousand litres of air. A grain of sand weighs about 10 µg. (**a**) The solid blue line represents measurements made on you during the study. You can compare your levels to other people in the study. Each square/dot on the chart represents a measurement made on another participant in Guatemala/Rwanda. Red dots are households that used a gas stove as part of the study. Green dots are households that used traditional stoves as part of the study. During the pre-intervention period, all households used traditional stoves. The solid black line is the recommended guideline exposure level established by the WHO during the study. The blue bars represent the study-wide average value for households like yours. (**b**) The red bars represent findings from measurements made on you. The solid black line is the recommended guideline exposure level established by the WHO. (**c**) The red circle represents the exposure level of your household. The green circle represents the study-wide average among households in your group (control vs intervention). The blue circle is the recommended guideline exposure level established by the WHO. The size of the red circle is your relative exposure on measurement days during this study.” (**d**) The final ‘thermometer’ graphical display used to depict PM_2.5_ exposure. Red colour at the left of the scale indicates less desirable exposure levels, while the green colour at the right indicates a cleaner or more desirable exposure level. Individual exposure is shown via the ‘open fire’ marker (for exposure before liquefied petroleum gas (LPG) intervention in the case of intervention households and for baseline exposure in the case of control households), and via the ‘gas cylinder’ marker (for exposure after LPG intervention in the case of intervention households).

The purpose of the DWG was to ask participants to interpret three different graphical representations of PM_2.5_ personal exposure data generated by HAPIN exposure scientists. Based on a ‘fictive narrative’, a kind of situational analysis, participants were asked to imagine that they were ‘LPG advocates who want to graphically display how to reduce air pollution in their homes’. Text about the graphical display in figure 2 was read verbatim to the participants, who discussed what they saw in each of these visual representations. We asked questions about specific words and elements in the graphical display. Based on their feedback, additional visual representations were produced and were presented again and discussed with participants, until a final representational model was mutually agreed on to be comprehensible and acceptable to participants in these settings.

The process was documented using worksheets that each group completed. On average, groups needed 70 min to review the three graphical displays of air pollution data. One person in each group read the text, and after the discussion, wrote down the results. If there was no agreement, we asked them to write down all answers given. If they did not understand the content, they were instructed to write ‘we don’t understand this word or this graph’.

**Figure 3:**
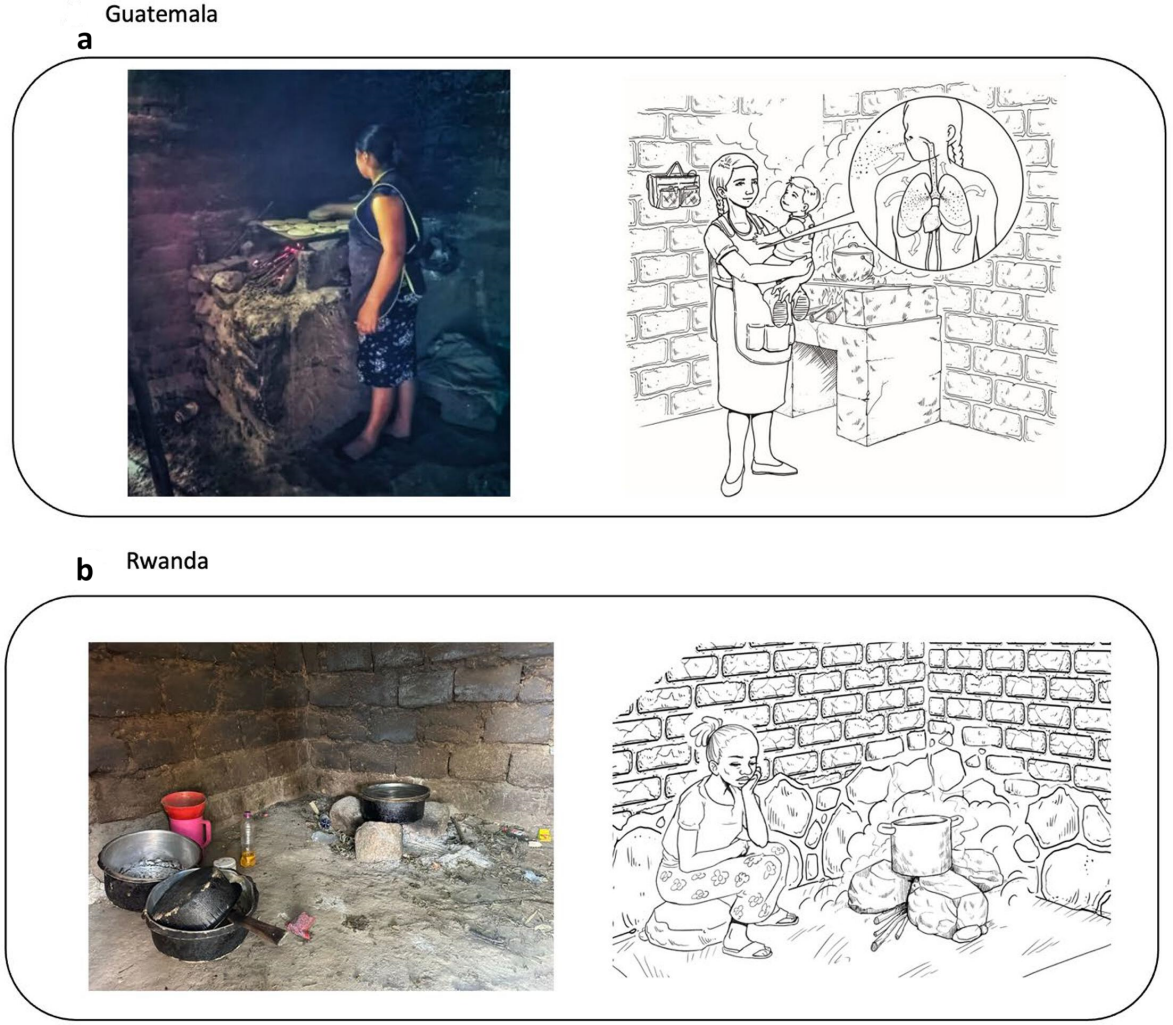
Artistic adaptations of household observations in Guatemala (a) and Rwanda (b). The left side of each panel is a photograph taken during household observation, while the right side is an early iteration of an artistic adaptation based on the photograph and desired messages. The person depicted in the photo is not a patient. She is a research participant, and she signed an informed consent to allow for the photograph to be taken.

*Step 4: Mini-focus groups*. We developed visual educational messages based on themes that emerged from the data collected during the participant observations and open-ended individual interviews and used the mini-focus groups to evaluate these messages. We aimed to enhance participants’ understanding of the relationship between air pollution and health and to enhance their environmental health literacy.^1 23^ In Guatemala, eight groups of three or four people (total n=32) assessed the prototype design (see figure 3). In Rwanda, we conducted four mini focus groups with 13 participants. The research team worked iteratively with a Guatemalan graphic designer who had prior experience working with the HAPIN team^24^ to develop images that depicted the study participants in settings similar to their own homes. HAPIN fieldworkers in Rwanda determined that the design and messages that emerged from the mini-focus groups in Guatemala would be equally meaningful in Rwanda. As a result, they decided to modify the visual images to represent their local environment.

Because we anticipated that few HAPIN participants would be able to continue cooking exclusively with LPG in the post-trial period, we focused on messages that portrayed a realistic set of activities that could feasibly reduce exposure even if cooking with biomass fuels continued to occur.

### Data analysis

Quantitative data from the environmental health literacy survey were summarised at the group level and reported as the number and percent of respondents agreeing with each binary statement. For PM_2.5_ air pollution data, we calculated descriptive statistics for control and intervention groups by study phase (baseline vs postintervention measures) and by country. Given the small sample size and right-skewed distribution of data, we used the Wilcoxon rank-sum test to evaluate the median differences at baseline and post-baseline by study arm and stratified by country.

Qualitative data were collected using audio recordings, open-ended questionnaires and photographs. Spanish interviews were transcribed verbatim and thematically coded using Nvivo by an anthropologist on our team. For interviews conducted in Kinyarwanda, transcripts were transcribed, translated into English and thematically coded by the Rwandan fieldworkers and the anthropologist using Excel, which was more accessible and feasible for them to use than Nvivo. The fieldworkers were trained to code in Excel and supervised by the anthropologist. Qualitative data were analysed using open coding, extracting themes that emerged from the data, and then grouped into categories. From these codes and categories, we used an inductive method to derive themes that were used to represent messages and images used in the posters. Photographs were used to develop the visual representations of findings from the study.

### Patient and public involvement

We did not involve patients in the design, conduct, reporting or dissemination plans of this research. Laura Ruiz Aguilar is a graphic designer and a member of the public who worked with us to co-produce the report-back materials produced for both Guatemala and Rwanda. Community-dwelling research participants were involved in the co-production of the report-back materials described here.

## Results

Results of the environmental health literacy survey conducted among 37 study participants in Guatemala suggested that participants on the whole were highly aware of the issue of air pollution exposure and believed that exposure was connected to health, providing consistently positive responses to questions designed to address the *Awareness and Knowledge* dimension of environmental health literacy as conceptualised in Gray.^23^ For example, 34 respondents (92%) agreed with the statement ‘In my community we have a problem with smoke in the air’; 100% agreed that ‘Smoke in the air can make people sick’ and 100% agreed that ‘Children who spend more time in the kitchen while their mothers are cooking have more health problems because of the smoke in the kitchen air’.

The types of health problems most associated with exposure to smoke tended toward acute symptoms like cough (n=30, 81%), headache (n=26, 70.2%), eye irritation (n=18, 48.6%) and dizziness (n=5; 13.5%) instead of chronic diseases like high blood pressure (n=3; <1%) and heart problems (n=2; <1%). Pneumonia (n=25; 67.6%), one of the primary outcomes of the HAPIN trial, was the most frequently mentioned disease. One-fifth (n=8; 21%) of the participants could not name a health problem associated with smoke exposure.

The results for responses intended to assess participants’ *Skills and Self-efficacy*, or their ability to enable health-protective decisions around reducing exposure to air pollution, were mixed. Although all respondents agreed that ‘I can make decisions that improve my health and the health of my family’ and 30 (81%) agreed that ‘Even when I cook with a wood-burning stove, there are things I can do to reduce the smoke in the air’, only slightly more respondents disagreed than agreed with the statement ‘The smoke that is produced when cooking with an open fire is a necessary part of life and there is not much we can do about it’: disagree—19 (52%) versus agree—15 (42%). 29 (78%) respondents agreed that gas stoves were too expensive to use all the time. 11 respondents, or 30% of the sample, said they ‘didn’t know’ how they could reduce smoke exposure while cooking.

The results related to *Community change* suggested that engagement of Guatemalan HAPIN participants with others in their community on issues related to air pollution was not common, particularly outside of immediate friends and family. Although 21 (57%) respondents ‘sometimes’ talk to friends and neighbours about the problem of air pollution, 14 (38%) ‘never’ did. Meanwhile, 36 (97%) ‘never’ talked to community leaders about these problems, and a majority (n=31, 84%) either responded ‘disagree’ or ‘don’t know’ to the statement ‘If there is something causing smoke in the air in my community, my neighbours and I act to get it stopped’.

### Activities to reduce exposure to household air pollution

Qualitative data from the household observations, interviews, working groups and mini-focus group discussions with HAPIN study participants resulted in the following potential exposure-reducing activities in each of the two settings:

#### Guatemala

- Use small pieces of wood (when cooking on a wood stove).
- Use a wood stove with a chimney.
- Open doors and windows when using a wood stove.
- Use a gas stove instead of a wood stove, if possible.
- Put trash in garbage sacks (rather than burning it).
- Take care of trees (rather than cutting them down for firewood).
- Recycle (rather than burning trash).

#### Rwanda

- Place your stove in a well-ventilated area.
- Protect your stove from wind and rain.
- After cooking, make sure the fire is out completely.
- Avoid using green wood or leaves, as these produce a lot of smoke.
- Avoid spending too much time in front of the stove, and keep babies and children away from the stove while cooking.
- Avoid blowing on the fire directly.
- Use a gas stove, if possible.

These messages, along with corresponding setting-specific visual images, were incorporated into the report-back materials.

### Personal exposure results

In Guatemala, there was no statistically significant difference in baseline PM_2.5_ exposures between control (n=19) and intervention (n=22) groups (Wilcoxon rank-sum, p=0.75). However, the differences in mean PM_2.5_ exposures after baseline were significantly different in the control (median 154 µg/m^3^, IQR: 93–197) and the intervention arms (median 21 µg/m^3^, IQR: 15–43) (Wilcoxon rank-sum, p<0.001). Similarly, in Rwanda, there was no statistically significant difference in baseline PM_2.5_ exposures between control (n=10) and intervention (n=10) groups (Wilcoxon rank-sum, p=0.10). However, the differences in mean PM_2.5_ exposures after baseline were significantly different in the control (median 88 µg/m^3^, IQR: 56–153) and the intervention arms (median 38 µg/m^3^, IQR: 24–48) (Wilcoxon rank-sum, p<0.003) (table 1).

**Table 1:** Personal 24-hour PM_2.5_ exposure levels among pregnant women in Guatemala and Rwanda (n=62) participating in the report-back pilot study

	Guatemala				Rwanda			
	Interventionn=22		Controln=20		Interventionn=10		Controln=10	
	Mean (SD)	Median(IQR)	Mean (SD)	Median(IQR)	Mean (SD)	Median (IQR)	Mean (SD)	Median(IQR)
PM_2.5_ (μg/m^3^)								
Baseline, <20 weeks	162 (113)	147 (63–251)	225 (392)	133 (77–162)	108 (85)	93 (48–132)	189 (112)	202 (98–258)
24–28 weeks	35 (39)	21 (15–46)	140 (81)	117 (76–186)	43 (28)	33 (23–53)	97 (53)	111 (45–141)
32–36 weeks	26 (16)	20 (11–42)	171 (117)	144 (74–262)	33 (17)	24 (23–43)	91 (33)	85 (71–115)
Pregnancy mean, after baseline	30 (25)	21 (15–43)*	155 (76)	154 (93–197)*	42 (26)	38 (24–48)†	90 (40)	88 (56–131)†

Difference in between-group medians after baseline.

* Wilcoxon rank-sum, p<0.001.

† Wilcoxon rank-sum, p<0.003.

Participants in the DWGs in both Guatemala and Rwanda had difficulty interpreting the three graphical representations of personal exposure data that had been developed by the study team (see figure 2). In contrast to the high-income settings where most work on environmental research report-back has occurred, participants in the HAPIN trial had notably lower levels of literacy on average (48% of Guatemalan and 42% of Rwandan women enrolled in the HAPIN trial had no formal education or had not completed primary school), and less familiarity with graphs and charts that are common means of visually representing scientific material. Despite the scripted guides provided by local, trained fieldworkers about what particulate matter is and how it is measured, participants expressed that they had trouble understanding what was being represented by the various bars, circles and dots on the graphs. They also had difficulty understanding the concept of comparing results (averaged) across all households in the study with their results.

Some of the main points of confusion for participants were: lack of understanding about what is meant by an ‘average’ for the household and study-wide (height of bars in figure 2b, red and green rings in figure 2c); lack of understanding about the reference value, specifically the WHO guidelines (black line in figure 2a, blue ring in figure 2c). In figure 2a, participants also had difficulty with the concept that each dot represented a different study household’s measurement and that the display of all the dots could be used to compare a given household’s measurements (blue lines) with measurements taken in other households participating in the study. While all understood the harm that is caused by exposure to ‘smoke’, the general concept of measuring particulate matter numerically (as particles per quantity of air) was also not easy for the study participants to comprehend.

After several rounds of discussion, it was decided that the visual materials depicting personal exposure to PM_2.5_ needed to relate to some form of visual representation of numeric data that was more familiar to participants. The researchers came up with the idea of the thermometer, which is an instrument that is widely available in both study communities. A thermometer depicts a linear numeric scale from low to high, and study participants were familiar with interpreting numbers visually in this way. At the time of this study, the Guatemalan government had implemented a ‘traffic light’ system to indicate the risk of COVID-19 contagion in Guatemala. The red-yellow-green colours were, in turn, associated with measures needed to control the spread of COVID-19, such as recommendations for social distancing, limits on the size of community gatherings and travel restrictions. The research team combined this ‘traffic light’ colour scale with the linear ‘thermometer-style’ scale, resulting in visuals shown in figure 2d. A marker was placed on the thermometer to depict a particular participant’s measured exposure to particulate matter. For intervention participants, there were two markers—one, illustrated with an ‘open fire’ motif, showing exposure at baseline (before the LPG stove intervention), while the second marker, illustrated with an image of an LPG cylinder, showed the average of all personal exposure measurements taken after the introduction of the LPG stove. Because control participants did not receive an LPG stove intervention, their ‘thermometer’ only contained one marker, depicting the average of all personal exposure measurements taken during the trial (and illustrated with the ‘open fire’ motif). Although the placement of the markers was determined numerically, no numbers were shown on the eventual images. Instead, a ‘red-yellow-green’ colour continuum was used to indicate more vs less desirable locations on the thermometer scale.

### Posters

**Figure 4:**
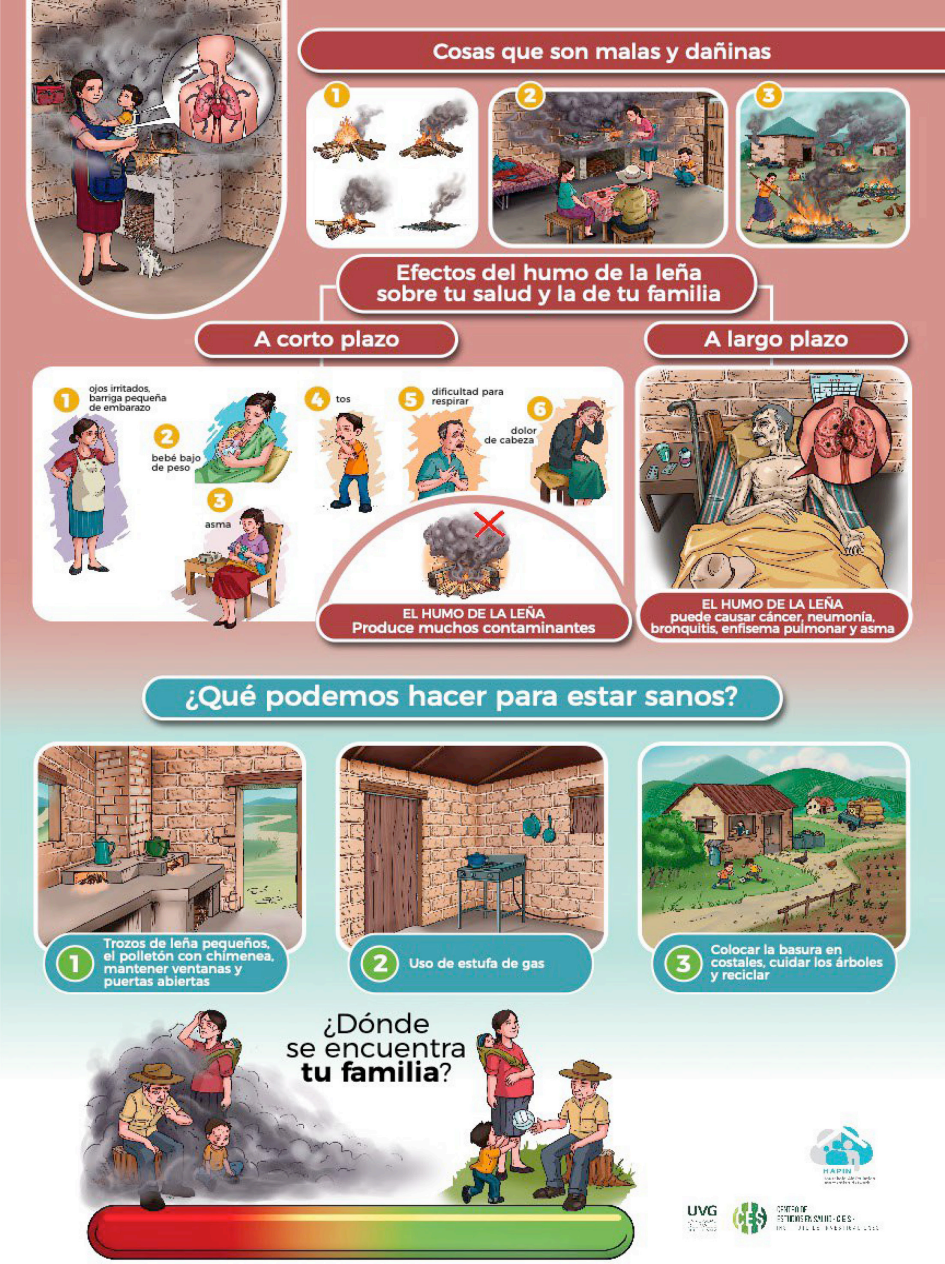
Report-back poster, Guatemala. Messages about the health effects of air pollution exposure and actions that can reduce exposure are shown. The participant-specific thermometer visual to depict individual PM_2.5_ exposure results would be included at the bottom.

The final step in the development of the report-back materials was the production of posters by a graphic designer. In each setting, posters were created that represented participant-curated messages about air pollution exposure, its health effects and actions that could be taken to avoid pollution. A space was left on each poster for a customised sticker that would present each household’s individual air pollution exposure results, using the community-preferred ‘thermometer’ graphic (figure 4).

Although education on the potentially harmful health effects of smoke was not an integral component of the HAPIN trial, participants in the report-back activities conveyed the importance of providing information related to the health effects of air pollution exposure. This health information was viewed by participants and investigators alike as important to contextualise the exposure results and to provide participants with the underlying reasoning ‘why’ air pollution exposure should be minimised. To address this aim, the graphic designer created images that visually represented what participants recognised as the irritative effects of smoke (watery eyes, headaches) as well as the chronic effects of exposure to air pollution, including adverse effects on their lungs and infant health, represented by low birth weight.

## Discussion

This is the first study, to our knowledge, that presents results on the development and application of communication methods to report back personal environmental exposure results to study participants in low-resource settings. Importantly, household air pollution from biomass used for cooking (eg, wood, crop waste, dung) was estimated to cause 3.2 million deaths in 2020^25^; dozens of stove intervention trials have been conducted to attempt to address this problem.^1326^,^32^ While some studies aimed to assess environmental health literacy about air pollution among village health volunteers in Thailand,^33^ environmental health literacy, respiratory symptoms and use of cookstoves in Kenya^34^ and calendar messages to promote health literacy to support the use of the LPG stove in the HAPIN trial,^24^ this is the first study to explore reporting-back of individual results to study participants who are exposed to this ubiquitous, high source of pollution.

The hurdles presented by such research in these settings—including low levels of literacy and numeracy—are not unique to low-income countries, however. Presenting complex scientific material to research participants in accessible and meaningful ways is a challenge even in high-income settings, and it is important for researchers to engage with communities to ensure that study results are presented in ways that are understandable and actionable. In a 2018 workshop that used a concept mapping exercise to identify key issues pertinent to reporting back environmental health research results,^35^ ‘Effective communication strategies’ was the topic rated as most important, followed by ‘Community knowledge and concerns’. The combination of community-engaged methods that were deployed in the report-back effort among HAPIN participants in Guatemala and Rwanda was designed to co-create meaningful and actionable messages reporting on air pollution exposure data in these communities, which was a key output of the HAPIN trial. These activities also highlighted the difficulty inherent in making complex scientific data visually accessible to a lay audience.

Although funding limitations restricted our activities to only two of the four HAPIN study settings on a pilot scale, and this small study limits generalisability of our findings, the methods and processes employed here could easily be replicated in other study settings. Longer-term follow-up to evaluate the effect of receiving the report-back materials on outcomes like pollution avoidance and engagement with the community on issues related to pollution would be a useful addition to future report-back efforts.^36^

As the activities described above make clear, developing report-back materials in a contextually appropriate and community-engaged way certainly requires expertise and significant staff time and cost. A limitation of this study was that we were unable to evaluate the report-back materials and the effect that these might have had on sustained behaviour change across all HAPIN participants. The benefits, if sustained, however, can be substantial. Report-back can enhance participants’ and communities’ ability to make desired changes to protect their health and the health of their families. In a study on childhood lead exposure in New York, reporting back of lead exposure information led some participants to make changes to reduce exposures, for example, by adapting improved cleaning behaviours and even changing residences.^37 38^ The information provided to study participants was seen as an important first step toward community mobilisation, and integral to the implementation of policies to reduce childhood lead exposure at a larger scale, such as regulations requiring proactive inspection and correction of lead hazards.^39^ Although few similar studies focused on personal measures of air pollution exposure have been reported, a study in Ohio developed report-back materials of personal air pollution monitoring with adolescents and their caregivers, using qualitative methods similar to ours, to develop electronic interactive maps using time-series data that was presented to study participants and their caregivers to guide strategies to reduce their exposures.^40^

Return of study results may also motivate people to participate in research and may ensure a more informed engagement with the research. For example, a survey among women who volunteered to be contacted about breast cancer studies found increased interest in participating in environmental studies if personal results would be returned.^41^ Reporting back of individual exposure information was shown to promote learning across a spectrum of contexts (eg, personal, sociocultural) that enhanced participants’ environmental health literacy.^42 43^ For example, receiving study results was shown to improve participants’ ability to seek out, comprehend and evaluate environmental health information; to make informed choices; and to reduce health risks.^1^ Engaging with research participants to interpret and translate study findings is also a means to ensure that participants’ health priorities are recognised, and to mitigate against the possibility of potentially harmful health consequences resulting from unintentional misunderstandings of study activities and findings (eg, from a recent cookstove trial, see Ardrey *et al*^44 45^).

Researchers and institutional review boards have expressed concerns about potential adverse effects of reporting back individual-level exposure data, including worries that results with uncertain clinical relevance might trigger anxiety or fear among participants, or that participants might be limited in their means to act to reduce harmful exposures and therefore frustrated by receiving results.^3^ These concerns, however, seem to play out infrequently. Researchers evaluating participant and provider reactions to report-back across a number of studies at the conclusion of the National Institute of Environmental Health Science-funded Personal Exposure Report-Back Ethics study concluded that “participants are almost universally eager to receive their results and do not regret getting them”.^2^ A study that investigated the attitudes of researchers and IRB representatives to report-back^46^ found that researchers who have implemented report-back overwhelmingly found that the benefits of report-back outweighed any pre-existing concerns. Even study participants who were limited individually in their ability to reduce exposures—for example, who lived near an active oil refinery—were able to use exposure information to mobilise action at the collective and judicial levels to protect community health. In this case, community action led to a court decision limiting the expansion of the refinery.^38^ A study reporting the results of a series of interviews with individuals involved in biomonitoring studies suggested that populations with low pre-existing levels of scientific literacy were just as interested in receiving their study results as more educated groups were.^47^ Further, low-literacy groups were not deterred by ambiguous results where the level of health danger was unknown, and appreciated receiving the ‘truth’ from scientists instead of a simpler and less complete answer.^48^ Reporting back environmental exposure information in an accessible and community-informed manner can thus be seen as a key lever towards enhancing environmental justice goals.^1^

Fortunately, developing the capacity to easily report back environmental exposure results to communities has been an ongoing effort in the environmental health community, and efforts are underway to encourage the mainstreaming of the practice. A handbook of report-back best practices and examples^9^ and a comprehensive set of recommendations from the National Academy of Sciences^3^ are two resources available to interested researchers. Further, digital tools such as the Digital Exposure Report-Back Interface (DERBI)^49^ are now available. Despite these laudable advances, however, participants in environmental health research studies in settings outside of high-income countries are largely being left out of report-back efforts. Although DERBI has been successfully adapted for environmental report-back in settings such as Massachusetts and Puerto Rico,^50 51^ its applicability may nonetheless still be limited in settings where constraints exist—for example, those with limited smartphone access and low levels of literacy, numeracy and familiarity with scientific visualisations. Given the numerous demonstrated benefits of reporting back individual environmental exposure information to study participants, we encourage the global and environmental health research community to make a greater effort to expand this practice in low- and middle-income country settings. The participants and communities contributing to scientific research in these settings deserve it.

## Conclusions

Development of materials to report back scientific results to study participants in accurate and understandable ways is an ethical obligation to communities participating in complex research and enables study participants to take action to reduce their exposure to harmful pollutants. Study participants in low-resource settings typically experience higher exposures to air pollution, yet there has been no evidence of report-back research done in these settings. In this report-back supplement to the HAPIN trial, a series of qualitative, quantitative and community-engaged activities were conducted to develop messages and materials to present personal air pollution exposure results to study participants in Guatemala and Rwanda. The processes documented here can serve as a model for other investigators to follow for the assessment of environmental health literacy and the development of community-informed report-back materials in their own study settings.

## Supplementary material

10.1136/bmjgh-2024-017672 online supplemental file 1

## Data Availability

Data are available upon reasonable request.
