# Supplementary material for: Development of personal air pollution exposure report-back materials to Household Air Pollution Intervention Network (HAPIN) trial participants in Guatemala and Rwanda: a qualitative study
Source: BMJ Glob Health. 2025 May 15;10(5):e017672. doi: 10.1136/bmjgh-2024-017672 (PMC12083286; doi:10.1136/bmjgh-2024-017672)
Supplement: online supplemental file 1 [file bmjgh-10-5-s001.pdf]

Supplement S1.
Report Back Environmental Health Literacy Survey Instrument

**Household ID Number:**<br><br>Número de identificación del hogar:
**Today's Date**<br>dd/mm/yyyy<br><br>**Fecha de hoy**<br>dd /mm /aaaa
**Who completed the form?**<br><br>**¿Quién completó el formulario?**

	**ASK:**<br>**PREGUNTAR:**	**RESPONSE:**<br>**RESPUESTA:**	**CODE:**<br>**CÓDIGO:**
1	What are the activities or things that produce smoke in the air? (mark all that apply)<br><br>¿Qué actividades o cosas producen humo en el aire? (marque todo lo que aplique)	1= car smoke<br>2= garbage smoke<br>3= cigarette smoke<br>4= woodsmoke from fire<br>5= agricultural burning to prepare for planting<br>6= other (specify)<br>888= don't know<br><br>1= humo del carro<br>2= humo de basura<br>3= humo del cigarro<br>4= humo de leña/fogón<br>5= roza de siembra<br>6= otro (especifique)<br>888= no se	
1a	If other, specify:<br><br>Si otro, especifique:	text<br><br>texto	
2	Do you agree or disagree with this phrase: "In my community we do not have a problem with smoke in the air."<br><br>Está usted de acuerdo o desacuerdo con esta oración: "En mi comunidad tenemos problemas de humo en el aire"	1= Agree<br>2= Disagree<br>3= Don't know<br><br>1= De acuerdo<br>2= En desacuerdo<br>3= No sé	

3	What health problems are caused by breathing smoke from an open fire/other biomass stove? (mark all that apply)<br><br>Cuáles son los problemas de salud que resultan por respirar el humo del fogón/poyetón/estufa de lena/plancha? (marque todo lo que aplique)	1= cough<br>2= headache<br>3= eye irritation<br>4= pneumonia<br>5= diarrhea<br>6= malnutrition<br>7= tired<br>8= high pressure<br>9 = heart problems<br>10= dizziness<br>11= other (specify)<br>888= don't know<br><br>1= tos<br>2= dolor de cabeza<br>3= irritación de ojos<br>4= neumonía<br>5= diarrea<br>6= desnutrición<br>7= cansancio<br>8= presión alta<br>9= problemas del corazón<br>10= mareos<br>11= otro (especifique)<br>888= no se	
3a	If other, specify:<br><br>Si otro, especifique:	text<br><br>texto	
4	Do you agree or disagree with this phrase: “Smoke in the air can make people sick.”<br><br>Está usted de acuerdo o desacuerdo con esta oración: "El humo en el aire puede causar enfermedades en las personas."	1= Agree<br>2= Disagree<br>3= Don’t know<br><br>1= De acuerdo<br>2= En desacuerdo<br>3= No sé	
5	Do you agree or disagree with this phrase: “Many people in my community have health problems because of the smoke in the air.”<br><br>Está usted de acuerdo o desacuerdo con esta oración: "Muchas personas en mi comunidad tienen problemas de salud por el humo en el aire."	1= Agree<br>2= Disagree<br>3= Don’t know<br><br>1= De acuerdo<br>2= En desacuerdo<br>3= No sé	
6	Do you agree or disagree with this phrase: The people in charge of cooking have more health problems because of the smoke in the kitchen air.	1= Agree<br>2= Disagree<br>3= Don’t know	

	Está usted de acuerdo o desacuerdo con esta oración: "Las personas encargadas de cocinar tienen más problemas de salud por el humo que se produce en la cocina."	1= De acuerdo<br>2= En desacuerdo<br>3= No sé	
7	Do you agree or disagree with this phrase: "Children who spend more time in the kitchen while their mothers are cooking have more health problems because of the smoke in the kitchen air."<br><br>Está usted de acuerdo o desacuerdo con esta oración: "Los niños que pasan más tiempo en la cocina mientras sus madres cocinan tienen más problemas de salud por el humo"	1= Agree<br>2= Disagree<br>3= Don't know<br><br>1= De acuerdo<br>2= En desacuerdo<br>3= No sé	
8	Do you agree or disagree with this phrase: "Even when I cook with an open fire, there are things I can do to reduce the smoke in the air/my exposure to the smoke in the air."<br><br>Está usted de acuerdo o desacuerdo con esta oración: "Los niños que pasan más tiempo en la cocina mientras sus madres cocinan tienen más problemas de salud por el humo"	1= Agree<br>2= Disagree<br>3= Don't know<br><br>1= De acuerdo<br>2= En desacuerdo<br>3= No sé	
9	What do you do to reduce the smoke while you are cooking? (mark all that apply)<br><br>¿Qué hace usted para reducir el humo mientras que está cocinando? (marque todo lo que aplique)	1= use gas<br>2= go outside the kitchen<br>3- open windows and doors<br>4= don't cook with green wood<br>5= other (specify)<br>888= don't know<br><br>1= usar gas<br>2= salir de la cocina<br>3= abrir ventanas o puertas<br>4= no cocinar con leña verde<br>5= otro (especifique)<br>888= no se	
9a	If other, specify:<br><br>Si otro, especifique:	text<br><br>texto	
10	Do you agree or disagree with this phrase: "The smoke that is produces when cooking with the open fire is a necessary part of life and there is not much we can do to avoid it."<br><br>Está usted de acuerdo o desacuerdo con esta oración: "El humo que se produce al cocinar con fogón es una parte necesaria de la vida y no podemos hacer mucho para evitarlo."	1= Agree<br>2= Disagree<br>3= Don't know<br><br>1= De acuerdo<br>2= En desacuerdo<br>3= No sé	
11	Do you agree or disagree with this phrase: "Even if I had a stove, it would be too expensive to use all the time."	1= Agree<br>2= Disagree	

	Está usted de acuerdo o desacuerdo con esta oración: “Aunque tuviera una estufa de gas, sería muy caro usarla todo el tiempo.”	3= Neither agree or disagree<br>4= Don’t know<br><br>1= De acuerdo<br>2= En desacuerdo<br>3=Ni de acuerdo o desacuerdo<br>3= No sé	
12	Do you agree or disagree with this phrase: “Even when I cook with an open fire, there are things I can do to reduce the smoke in the air.”<br><br>Está usted de acuerdo o desacuerdo con esta oración: “Aunque cocino con un fogón, hay cosas que puedo hacer para reducir el humo en el aire.”	1= Agree<br>2= Disagree<br>3= Neither agree or disagree<br>4= Don’t know<br><br>1= De acuerdo<br>2= En desacuerdo<br>3=Ni de acuerdo o desacuerdo<br>3= No sé	
13	Do you agree or disagree with this phrase: “I can make decisions that improve my health and the health of my family.”<br><br>Está usted de acuerdo o desacuerdo con esta oración: “Yo puedo tomar decisiones para mejorar mi salud y la de mi familia.”	1= Agree<br>2= Disagree<br>3= Don’t know<br><br>1= De acuerdo<br>2= En desacuerdo<br>3= No sé	
14	How often do you talk to your neighbors/friends about the problem of air pollution?<br><br>Con que frecuencia habla usted con sus vecinos y/o familia acerca del problema de humo en el aire?	1= Never<br>2= Sometimes<br>3= Always<br><br>1=Nunca<br>2= A veces<br>3= Siempre	
15	How often do you talk to community leaders about the problem of air pollution?<br><br>¿Con que frecuencia habla usted con los lideres de su comunidad acerca del problema de humo en el aire?	1= Never<br>2= Sometimes<br>3= Always<br><br>1=Nunca<br>2= A veces<br>3= Siempre	
16	Do you agree or disagree with this phrase: “When I talk to others about the problem of air pollution, we come up with new ideas for things we can do to make the problem better.”	1= We never have ideas<br>2= We sometimes have ideas<br>3= We always have ideas	

	Está usted de acuerdo o desacuerdo con esta oración: "Cuando hablo con personas sobre el problema de humo en el aire, surge nuevas ideas do como mejorar el problema."	4= I never talk with others about this<br><br>1= Nunca tenemos ideas<br>2= A veces tenemos ideas<br>3= Siempre tenemos ideas<br>4= Nunca hablo con otros sobre este tema	
16	Do you agree or disagree with this phrase: If there is something causing smoke in the air in my community, my neighbors and I act to get it stopped.<br><br>Está usted de acuerdo o desacuerdo con esta oración: "Cuando hablo con personas sobre el problema de humo en el aire, surge nuevas ideas do como mejorar el problema."	1= Agree<br>2= Disagree<br>3= Don't know<br><br><br>1= De acuerdo<br>2= En desacuerdo<br>3= No sé	
17	Can you describe to me the purpose of the HAPIN trial? (mark all that apply)<br><br>¿Me puede decir cuál fue el propósito del estudio HAPIN? (marque todo lo que aplique)	1= measure air pollution<br>2= child pneumonia<br>3= child weight<br>4= child height/growth<br>5= child development/behavior<br>6= child health (not specified)<br>7= blood presser of the adult woman<br>8= adult woman health (not specified)<br>9= health of the pregnant women<br>10= other (specify)<br><br>1= medir humo en el aire<br>2= neumonía del niño 3= peso del niño<br>4= talla/crecimiento del niño<br>5= desarrollo/ comportamiento del niño<br>6= salud (no especifica) del niño<br>7= presión arterial de mujer adulta<br>8= salud (no especifica) de la mujer adulta	

		9= salud de la mujer embarazada<br>10= otro (especifique)	
	If other, specify:<br><br>Si otro, especifique:	text<br><br>texto	
